# Central Nervous System Hemangioblastoma in a Pediatric Patient Associated With Von Hippel-Lindau Disease: A Case Report and Literature Review

**DOI:** 10.3389/fonc.2021.683021

**Published:** 2021-05-24

**Authors:** Bo Yang, Zhenyu Li, Yubo Wang, Chaoling Zhang, Zhen Zhang, Xianfeng Zhang

**Affiliations:** ^1^ Department of Neurosurgery, First Hospital of Jilin University, Changchun, China; ^2^ Department of Pediatrics, First Hospital of Jilin University, Changchun, China

**Keywords:** central nervous system, Von Hippel-Lindau disease (VHL disease), hemangioblastoma, pediatric neurosurgery, literature review

## Abstract

**Background:**

Hemangioblastoma is a benign tumor of the central nervous system and may appear as a component of von Hippel-Lindau (VHL) disease. At present, approximately 40 cases of optic nerve HGBs have been reported in the literature. VHL disease is a rare autosomal-dominant inherited cancer syndrome with different phenotypes caused by variants in the VHL gene. Herein, the authors describe a case of a pediatric patient with VHL disease and with optic nerve HGB, a rare phenotypic expression. The purpose of this study was to explore the genotype-phenotype, clinical features, treatment and follow-up of VHL-associated hemangioblastomas in pediatric patients.

**Case Description:**

A 12-year-old boy presented with vision loss, headache and dizziness at our hospital. Magnetic resonance imaging (MRI) revealed a large (19.8 mm*18.5 mm*23.5 mm) irregular mass located in the suprasellar region. The mass was successfully removed after craniotomy and microsurgical treatment. The pathological diagnosis was left optic nerve HGB. Genetic analyses showed p.Pro86Leu (c. 257C>T) heterozygous missense mutations in the VHL gene.

**Conclusion:**

This is the first reported pediatric case of VHL-associated optic nerve HGB. The genotype-phenotype correlation of VHL disease may provide new evidences for predicting tumor penetrance and survival. Gross tumor resection combined with stereotactic radiosurgery might be the most beneficial treatment.

## Introduction

Hemangioblastoma (HGB) of the central nervous system (CNS) is a rare indolent vascular tumor characterized as a benign, slow-growing, non-metastasizing neoplasm, representing 2% of cranial tumors (1, 2). HGB is even rarer in pediatric patients, with an incidence of less than 1 per 1,000,000 (3, 4). HGBs of the CNS are commonly benign, but they may lead to significant irreversible neurological deficits and even severe disabling morbidities based on their location and multiplicity. Approximately 30% of HGBs are associated with von Hippel-Lindau (VHL) disease and are often multifocal, situated in the spinal cord, cerebellum and brainstem (1, 5, 6). HGBs in the sellar region or suprasellar region are relatively uncommon, and optic nerve HGBs are extremely rare, with approximately 40 reports in the literature, including this case (7–9).

VHL disease is a rare autosomal dominantly inherited cancer syndrome. It is associated with a mutation in the VHL tumor suppressor gene, which is located on chromosome band 3p25-26 (10, 11). Once pathogenic variants occur, patients can exhibit a series of clinical manifestations: CNS HGBs, pheochromocytomas, clear cell renal cell carcinomas (RCCs), renal cysts, pancreatic cysts, pancreatic neuroendocrine tumors, endolymphatic sac tumors and so on (10, 12). Among them, pheochromocytomas may be the only and/or initial manifestation in pediatric patients with VHL disease, with delayed manifestation in other organs; the presence of CNS HGBs as the first clinical manifestation of VHL disease is extremely rare. The risk of VHL disease in patients with CNS HGBs is negatively correlated with patient age (13), illustrating the importance of genetic testing in pediatric patients with HGBs.

Here, we describe the first VHL-associated optic nerve HGB in pediatric patients, a rare phenotypic expression. We also provide a literature review of VHL-associated HGBs in pediatric patients to summarize the genotype-phenotype, clinical features, treatment and follow-up of this rare disease in this population.

## Case Report

**Figure 1:**
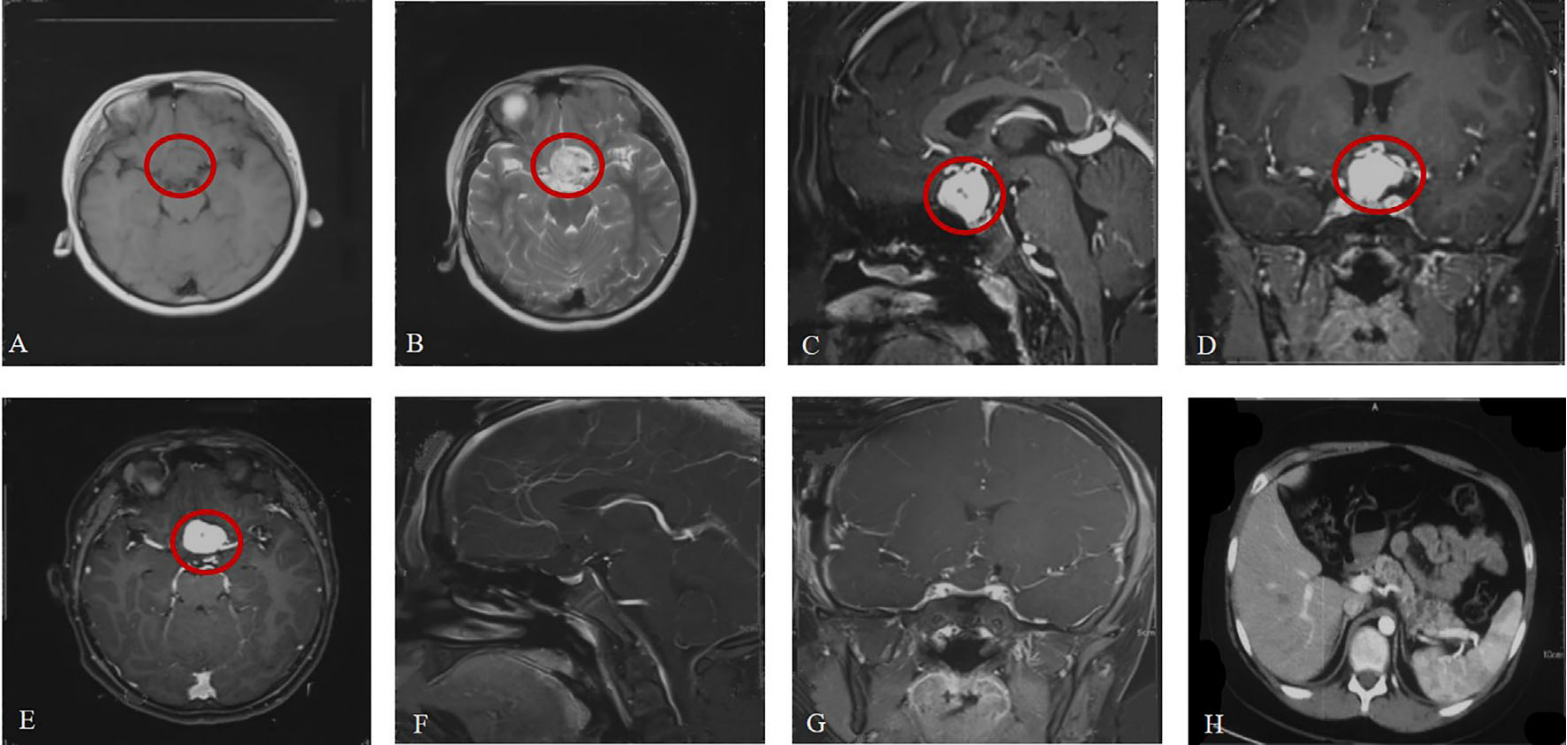
Brain MRI at admission **(A–E)**. **(A, B)** Axial view (weighted sequence in T1, T2). **(C–E)** Sagittal, coronal, axial view after contrast injection, with the tumor marked. **(F, G)** Brain MRI after surgery. **(H)** Pancreatic cysts.

A previously healthy 12-year-old boy was admitted to the hospital with vision loss, headache and dizziness over 1 month, which was aggravated in the last 5 days. Magnetic resonance imaging (MRI) of the head revealed a large (19.8 mm*18.5 mm*23.5 mm) irregular mass located in the suprasellar region abutting the left aspect of the optic chiasm. It appeared isointense on T1-weighted imaging (T1WI) and homogenously hyperintense on T2-weighted imaging (T2WI). After gadolinium administration, a well-circumscribed enhancing lesion was observed (**Figures 1A–E**). A small cerebellar lesion was also confirmed.

The mother of the patient underwent surgery for HGB in the CNS (spine, cerebellum) and had pheochromocytomas and pancreatic cysts. On the basis of the mother’s medical history, clinical manifestations and imaging examinations, she was diagnosed with VHL disease, although she refused genetic sequencing. Therefore, the patient was examined for VHL disease before surgery. Sequencing of exon 1 of the VHL gene revealed a c.257C>T mutation (amino acid p.P86L). Ophthalmological examination revealed that the boundary of the optic disc in the left eye was pale and that there were no abnormalities in the right eye. Abdominal computed tomography (CT) was also performed to identify associated lesions and pancreatic cysts (**Figure 1H**). Based on these findings, the patient was definitely diagnosed with VHL syndrome.

**Figure 2:**
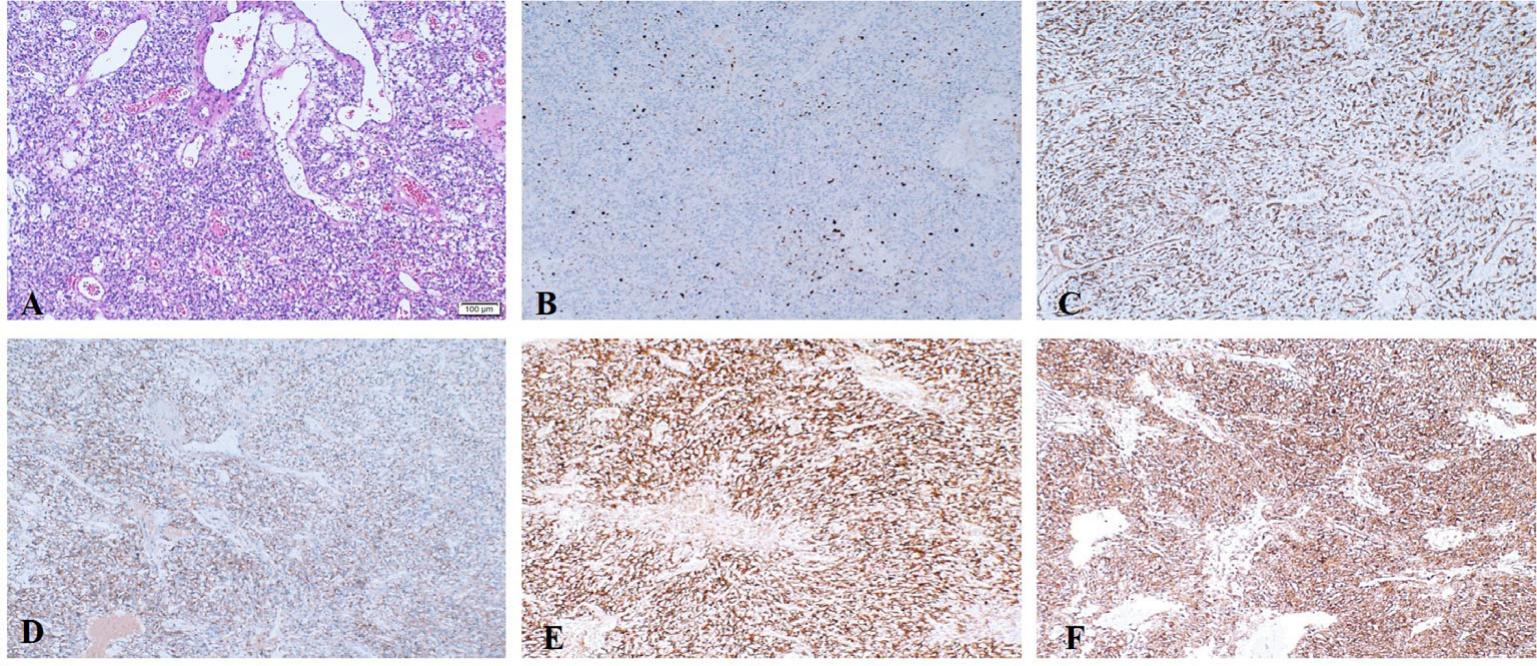
Representative postoperative pathological images. **(A)** Histologic examination of optic nerve HGB. **(B–F)** Immunohistochemical staining. **(B)** The proliferative fraction of tumor cells (Ki-67) was low, at approximately 2%. **(C)** CD-34 showed abundant blood vessels. Tumor cells showed positivity for EGFR **(D)**, NSE **(E)** and vimentin **(F)**.

**Figure 3:**
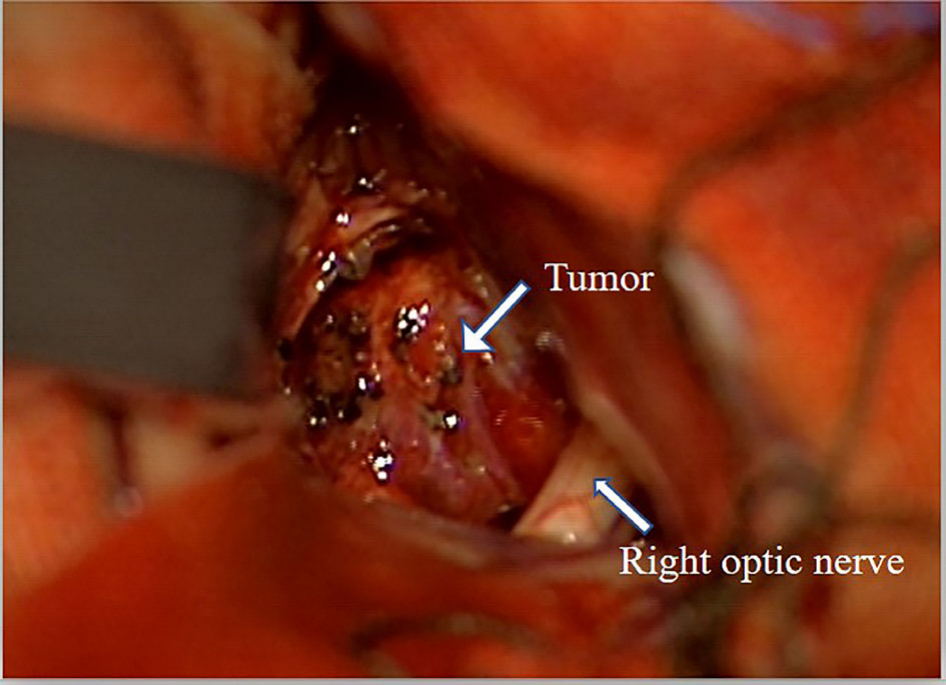
Surgical pictures. Severe adhesion of the tumor and left optic nerve.

He underwent surgery to remove the tumor from the suprasellar region at our neurosurgical department, and histology confirmed the diagnosis of HGB (**Figures 2** and **3**). The cerebellar lesion was not in the same surgical field as the suprasellar tumor and had no obvious mass effect, so they were not treated at the same time. Immunohistochemical analysis indicated that the Ki-67 index was approximately 2%. Furthermore, the neoplasm was positive for EGFR, NSE, CD34 and vimentin and negative for CK-pan, Pax-8, CD10, CD56, S-100, glial fibrillary acidic protein, inhibin, SSTR2, and epithelial membrane antigen (**Figure 2**).

The patient was transferred to the neurosurgery intensive care unit for postoperative monitoring and was ambulatory on postoperative day 1. Postoperative MRI (**Figures 1F, G**) demonstrated that the lesion in the suprasellar region was completely removed. He was discharged on postoperative day 7, and regular follow-up was performed. Unfortunately, the patient lost vision completely in the left eye after surgery.

## Discussion

Here, we reported a pediatric case of VHL disease presenting with HGBs in the optic nerve and cerebellum and pancreatic cysts. The diagnosis of VHL disease was confirmed by the identification of a familial germline mutation located in the first exon of the VHL gene (c.257C>T, p.P86L), which has been reported in the Human Gene Mutation Database and VHLdb databases (14, 15). To date, only 7 pediatric cases of sellar or suprasellar HGB (including optic nerve HGBs) have been reported (**Table 1**). Regarding suprasellar HGBs, the pituitary stalk may be the site of origin in the vast majority of cases and, rarely, the intracranial portion of the optic nerve. In our case, we confirmed during surgery that the tumor was mainly located inside the optic nerve without a discernible tumor capsule and without the involvement of the leptomeninges. Given the rarity of suprasellar VHL-associated HGBs in childhood, we review the genotype-phenotype association of VHL disease, treatment, natural history and follow-up.

**Table 1:** Published pediatric reports of sellar or suprasellar HGB (including optic nerve HGB).

Author,Year	Age at first manifestation/Sex	Country	First manifestation	Original site in the CNS	Recurrent site in the CNS	Interval to recurrence	Treatment	Other sites(excluding the CNS)	GeneticVHL	Mutation description	Clinical VHL	Clinical type
Lee, 2013	15/M	Korea	intermittent headache, vomiting	cerebellum	pituitary, cervical spine, thoracic spine	7y	suboccipital craniotomy; radiotherapy	RCC, kidney cysts, pancreatic cysts	No	No	Yes	1A
Ajlerp, 2012	12/F	Argentina	Headache, bitemporal hemianopsia	sellar	No	NA	transsphenoidal craniotomy;	No	No	No	No	-
Lonser, 2009	11-18y	USA	Asymptomatic	Pituitary stalk	NA	NA	conservative manner	NA	Yes	NA	Yes	-
Meyerle, 2008	15y/F	USA	Asymptomatic	Optic nerve	No	NA	transnasal–transsphenoidal excision	retina	Yes	Nonsense mutation	Yes	1B
Kouri, 2000	15/F	USA	Asymptomatic	Optic nerve	No	NA	transsphenoidal craniotomy;	retina	NA	NA	Yes	1B
Sawin, 1996	11y/F	USA	Headache, progressive visual loss	Sellar	No	1y	subfrontal craniotomy; radiotherapy	No	NA	NA	Yes	1B
Lauten, 1981	15y/M	USA	decreasing visual acuity, proptosis	Optic nerve	No	NA	Craniotomy	No	NA	NA	No	-
Our case	12/M	China	Headache, progressive visual loss	Optic nerve	NA	NA	Craniotomy	pancreatic cysts	Yes	c.257C>T p.P86L	Yes	1B

CNS, central nervous system; NA, not available; Pheo, pheochromocytoma; SRS, stereotactic radiotherapy; RCC, renal cell carcinoma.

### The Genotype-Phenotype Association of VHL Disease

The mutation types and locations of the VHL gene have been reported to be associated with certain phenotypes, suggesting the necessity of genetic sequencing in patients with suspected VHL disease.

Typical classification methods divide VHL patients into two types, depending on the absence or presence of phaeochromocytoma (1, 10). Type 1 patients have a higher lifetime risk of HGBs and a lower risk of phaeochromocytoma development; in turn, the risk of phaeochromocytoma in type 2 patients is estimated at approximately 40–60% (10, 16). Type 2 patients are subsequently subdivided into 3 subtypes based on the risk of RCC development: Type 2A (susceptibility to HGBs and pheochromocytoma but rarely RCC), Type 2B (susceptibility to HGBs, RCC, and pheochromocytoma), and Type 2C (susceptibility to pheochromocytoma only) (10, 11, 17).

Missense mutations and truncating mutations are the most common pathogenic variants in the VHL gene. Deletions and truncating mutations are more highly associated with VHL type 1 disease, while missense mutation carriers seem to have a higher risk of VHL type 2 disease (11, 18, 19). This association also leads to a higher risk of pheochromocytomas in patients with missense germline variants compared to those with truncating variants, a trend that is reversed for CNS HGBs, RCC and pancreatic cysts (11, 19–21). Notably, missense mutations appear to result in a milder phenotype than deletions or truncating mutations because the residual VHL protein can maintain intrinsic function (22–24).

The result of different germline mutations is VHL protein (pVHL) inactivation. The complex formed by pVHL and elongation factors functions as an E3 ligase, and the β-domain of pVHL binds directly to hypoxia-inducible factor-α (HIF-α) *via* residues 65–117 (1, 25, 26). The loss of functional pVHL results in the accumulation of HIF-α, which leads to the upregulation of endothelial growth factor (VEGF), platelet-derived growth factor-β (PDGF**-** β**)** and transforming growth factor-α (TGF-α) and eventually leads to tumorigenesis and the proliferation of microvascular vessels (10, 27, 28). Taken together, these findings may explain why patients with non-HIF-α binding site mutations seem to have a much better survival and prognosis than those with HIF-α binding site mutations (20, 29).

Due to the lack of relevant pediatric studies, the genotype-phenotype association of VHL disease come from the clinical data of adult patients (29–31). In our case, genetic analysis revealed a missense mutation in the VHL gene, and the patients’ clinical manifestation should be closer to that of VHL type 2 disease. However, he presented with symptoms of CNS HGBs and did not develop pheochromocytoma. Hence, the genotype-phenotype association of VHL disease in pediatric patients needs to be further summarized in a large sample size.

### Treatment

Although the optimal surgical timing for resection of VHL-associated CNS HGBs and the effect of radiotherapy and chemotherapy remain unclear, tumor resection is currently the mainstay of therapy for VHL-associated CNS HGBs. The standard surgical approach for CNS HGBs is craniotomy and microsurgical treatment, which has the advantages of versatility and applicability for repeated surgeries using the same strategy and the disadvantage of invasiveness (6, 10, 32). Endoscopic transcranial surgery, a novel and emerging operation, is minimally invasive, but it is not clear whether it can achieve the same level of efficacy as conventional craniotomy (33).

The optimal timing of surgical intervention remains unclear due to a lack of studies clarifying the surgical indications (6, 34, 35). A recent prospective long-term study of VHL patients showed that only 6.3% of all HGBs became symptomatic and required surgery during the study period (mean, 6.9 years), and the development of new tumors decreased with age (6, 24, 36). The study results suggested that consideration of surgical resection for symptomatic lesions and the avoidance of unnecessary treatments for asymptomatic tumors that may not progress can provide lasting clinical stability for most patients. We recommend that patients with HGBs, especially those with VHL-associated HGBs, require immediate surgery when CNS symptoms appear or when radiological characteristics of asymptomatic tumors indicate an increase in tumor volume.

Recently, stereotactic radiosurgery (SRS) has been investigated as an alternative treatment modality for CNS HGBs, and it is more suitable for patients with multiple or recurrent tumors and nonsurgical candidates (37, 38). When it comes to pediatric patients, the potential risk of late sequelae should not be ignored (32, 39). In addition, novel drugs have been developed based on the key pathways involved in HGB development, and their safety and effectiveness are being evaluated by several clinical studies (1, 6, 40, 41). These medications include antiangiogenic agents, anti-VEGF agents, histone deacetylase inhibitors and inhibitors designed for HIFs (6, 42, 43). Among them, antiangiogenic agents were reported to halt tumor growth in RCC, and anti-VEGF agents may be more suitable for retinal HGBs and CNS HGBs (44–46). In pediatric patients, the therapeutic drugs are promising approach to delay the onset of symptoms and thus delay surgery.

There are no definite guidelines concerning the treatment of CNS HGBs in the pediatric and adolescent age group. Surgical resection of HGBs in pediatric patients is considered to be more challenging than in adults due to the higher potential risks of massive bleeding and damage to adjacent functional tissue. In our case, preoperative embolization of multivascular brain tumor may be a good choice. In addition, the use of SRS and drug therapy in pediatric patients is still in the stage of empirical treatment due to the limited clinical data. We expect a large retrospective study to better summarize the treatment regimens for VHL-associated HGBs in pediatric patients.

### Natural Disease Course and Follow-Up

VHL disease is a long-term chronic disease that is characterized by a lifetime risk of multiorgan involvement, and approximately 72% of patients inevitably develop tumor recurrence or even new lesions (12, 24, 47). Sex has an effect on tumor development, as male hormones are associated with an increased CNS HGB burden and growth rate (24). Furthermore, multiplicity at the time of diagnosis and younger age at onset have been associated with an increased rate of tumor development, which usually portends adverse clinical outcomes (4, 12). Unexpectedly, it has been reported that the tumor location may also affect growth rate, as tumors in the medulla grow more slowly than those in the cerebellum or brainstem (20, 24).

There are three different patterns of CNS HGB progression: saltatory (72% of growing tumors), exponential (22%) and linear (6%) (5, 24, 34). The tumor may remain quiescent for a long time, so it is necessary to extend the follow-up period appropriately. In general, physical examination with neurological assessment is recommended every 6 months for the first 2 years after surgery in patients with a family history of VHL, followed by an annual check-up during subsequent years until the end of the follow-up period (19, 48). A reasonable and practical proposal may also consist of annual abdominal ultrasonography, a fundus examination and an audiometric evaluation. Karnofsky Performance Scale scores are usually used for the initial assessment of the clinical progression of CNS HGBs. During the teenage years, yearly abdominal ultrasonography should be performed, with particular attention paid to the kidneys, pancreas, and adrenal glands (16, 48). For children with positive family history or positive genetic testing for a VHL mutation, an extensive medical, radiological, urological, neurological and ophthalmological screening should be performed every year, regardless of whether they undergo surgery or not. Notably, although there is consensus regarding the need for lifelong follow-up, controversy remains over the type of imaging tests and the frequency of screening assessments.

## Conclusion

Optic nerve HGBs are exceedingly rare tumors associated with VHL disease. The genotype-phenotype correlation of VHL disease can be classified into truncating mutations and missense mutations, which may play an important role in predicting tumor penetrance and survival. The definitive treatment for these lesions is surgical resection, followed by SRS if necessary. When examining pediatric patients with suprasellar tumors, HGB should also be considered in the differential diagnosis; in addition, oncologists and surgeons should be aware that CNS HGBs in pediatric patients can be associated with VHL disease, and regular follow-up is mandatory. Given the high association between pediatric HGBs and VHL disease, we recommend that each pediatric patient with CNS HGBs should be screened for germline mutations of the VHL gene.

## Author Contributions

XZ design the study and perform the surgery. BY, ZL, YW, CZ, and ZZ collected the data. BY, ZL, and YW prepared the manuscript. All authors contributed to the article and approved the submitted version.

## Conflict of Interest

The authors declare that the research was conducted in the absence of any commercial or financial relationships that could be construed as a potential conflict of interest.
